# Design rules for bioorthogonal glycan-based tools

**DOI:** 10.1042/BCJ20260542

**Published:** 2026-09-18

**Authors:** Freya Hoddle, Sophie Dela Schmidt, Sandhya Sridhar, Benjamin Schumann

**Affiliations:** 1 Department of Chemistry, Imperial College London, W12 0BZ London, U.K.; 2 Chemical Glycobiology Laboratory, The Francis Crick Institute, NW1 1AT London, U.K.; 3 Faculty of Chemistry and Food Chemistry, TUD Dresden University of Technology, 01067 Dresden, Germany

**Keywords:** Bioorthogonal, carbon metabolism, Glycosylation, Glycosyltransferase

## Abstract

As one of the most abundant and diverse post-translational modifications in biology, glycosylation has a significant influence on the functions of proteins in health and disease. To untangle the complexity of glycan biosynthetic pathways, the process of studying glycosylation has been expedited by specialised, chemical labelling techniques. In the present review, we explore the development of metabolic glycan labelling from early biochemical methods to current approaches that use interdisciplinary techniques. We highlight work that has informed our understanding of glycan biosynthesis through pioneering chemical biology approaches. Moreover, we provide guidance for experimental design that can be tailored to the specific needs of those investigating glycosylation in the context of their own work. Our ‘Design Rules’ consolidate the findings of several groups in a simple roadmap to make metabolic glycan labelling as effective as possible.

## Introduction

Glycosylation is one of the most abundant post-translational modifications, and glycan modifications have a strong influence on the biological function of proteins. Unlike proteins, glycans are secondary gene products, and glycosylation is regulated by the complex interplay of over 200 glycosyltransferases (GTs) and glycosidases in humans. Due to this non-templated synthesis, the investigation of glycan modifications is not straightforward. Asn(N)-linked glycosylation is characterised by the addition of a glycan precursor to a common consensus sequence (Asn-Xaa-Ser/Thr, with Xaa any amino acid except Pro), although this sequon is being further refined [1]. Ser/Thr/Tyr(O)-linked glycosylation is more heterogeneous and generally has no consensus sequence, making it more challenging to study. Many cytoplasmic and nuclear proteins are modified by O-linked *N*-acetylglucosaminyl (O-GlcNAc) glycosylation. Intracellular O-GlcNAc glycosylation is characterised by the addition of a single GlcNAc onto a Ser or Thr residue by the UDP-GlcNAc:polypeptidyltransferase (OGT). O-linked *N*-acetylgalactosaminyl (O-GalNAc) glycosylation occurs in the secretory pathway by the addition of GalNAc onto a Ser, Thr or Tyr residue on protein substrates. The initiation of O-GalNAc glycosylation is catalysed by a family of 20 GTs in humans called polypeptide GalNAc transferases (GALNTs/GalNAc-Ts) [2–4]. Certain isoenzymes of the GalNAc-T family are expressed ubiquitously in the human body (e.g. GalNAc-T1, -T2) while others are restricted to certain tissues or cells, such as GalNAc-T9 in the brain [5–7]. Dysregulation of *GALNT* expression or mutations in these enzymes is associated with a range of pathologies, including cancer and SARS-CoV-2 infection [8,9].

Established approaches in molecular cell biology, such as knockout (KO) studies of genes encoding GTs, have been fundamental to our understanding of glycosylation pathways [10–12]. For instance, the finding that the chaperone Cosmc is important for extension of O-GalNAc glycans was followed by the genetic KO of the *COSMC* gene to facilitate glycoprotein analysis [13]. This ‘SimpleCell’ strategy facilitated the discovery of global O-glycosites, using zinc-finger nuclease-engineered *COSMC*-KO cell lines with simplified homogeneous O-glycans [14]. Subsequent studies leveraged this approach to explore the individual enzyme-substrate relationships amongst the GalNAc-T family. Schjoldager et al. showed that by generating isogenic variants of the HepG2 hepatocellular carcinoma cell line with KOs of GalNAc-T1 and -T2, it was possible to quantitatively compare glycosylation site occupancy through lectin enrichment and LC-MS/MS analysis [15]. While most glycosylation events are redundant, isoform-specific patterns were identified and confirmed with *in vitro* glycosylation assays. Furthermore, in the same study, specific GalNAc-T enzymes were shown to be associated with specific biological pathways, suggesting more functional implications of each isoenzyme. The way in which the isoenzymes orchestrate regulation amongst redundant substrates was further explored by Hintze and colleagues [16]. By using HEK293 cell lines with Tet-On inducible expression of *GALNT2* and *GALNT11*, dose-dependent glycosylation of isoform-specific glycosites was described with quantitative O-glycoproteomic approaches. The specificity of GalNAc-T enzyme-substrate relationships is important in understanding the role of isoenzymes in disease contexts. GALNT2 was implicated as a regulator of high-density lipoprotein-C metabolism. Work by Khetarpal et al. found that loss of function of GALNT2 has an influence on phospholipid transfer activity and protein processing, by identifying T2 glycosylation sites on phospholipid transfer proteins [17]. Yang and colleagues also investigated the role of GALNT2 in congenital disorders of glycosylation (CDG). A GALNT2-deficient mouse model was used to determine changes in glycosylation occupancy compared with a wild-type mouse by quantitative proteomics. Mapping of GALNT2-specific O-glycosites in proteins in mouse liver revealed that tissue-specific changes occurred with CDG of GALNT2, with mechanisms linked to changes in metabolism [18]. Similarly, in a *Galnt3-/-* mouse model, providing a recapitulation of GALNT3-CDG, mass spectrometry (MS) analysis revealed hundreds of GALNT3-specific glycosites relevant to disease biology [19].

While powerful, approaches in molecular cell biology can be limited by the complex interplay between GTs in the secretory pathway, for instance through substrate redundancy and compensatory activities. A widely used approach to study glycan modifications is metabolic oligosaccharide engineering (MOE). This method introduces a chemical modification into monosaccharides that are taken up by cellular biosynthetic pathways and incorporated into a range of glycoproteins. The modification typically includes a bioorthogonal handle that can be reacted with reporter moieties such as biotin or fluorophores [20,21]. MOE allows the detection and identification of glycan modifications using various methods, such as imaging and MS. Chemists and biologists use the strategy as a common tool to investigate cellular glycosylation as a complement to genome engineering. Following pioneering work by Reutter, MOE was first developed for sialic acids (Sia) and has since been expanded to other monosaccharides [22–24]. Analogues of GalNAc and GlcNAc have rapidly emerged as some of the most important MOE reagents, some of which are still being heavily used more than 20 years later. Over the years, our understanding has evolved of how these tools are best designed and used in cell biology. Here, we aim to give a guide to the design rules for MOE to study different types of glycosylation.

## Metabolic oligosaccharide engineering on sialic acids

**Figure 1:**
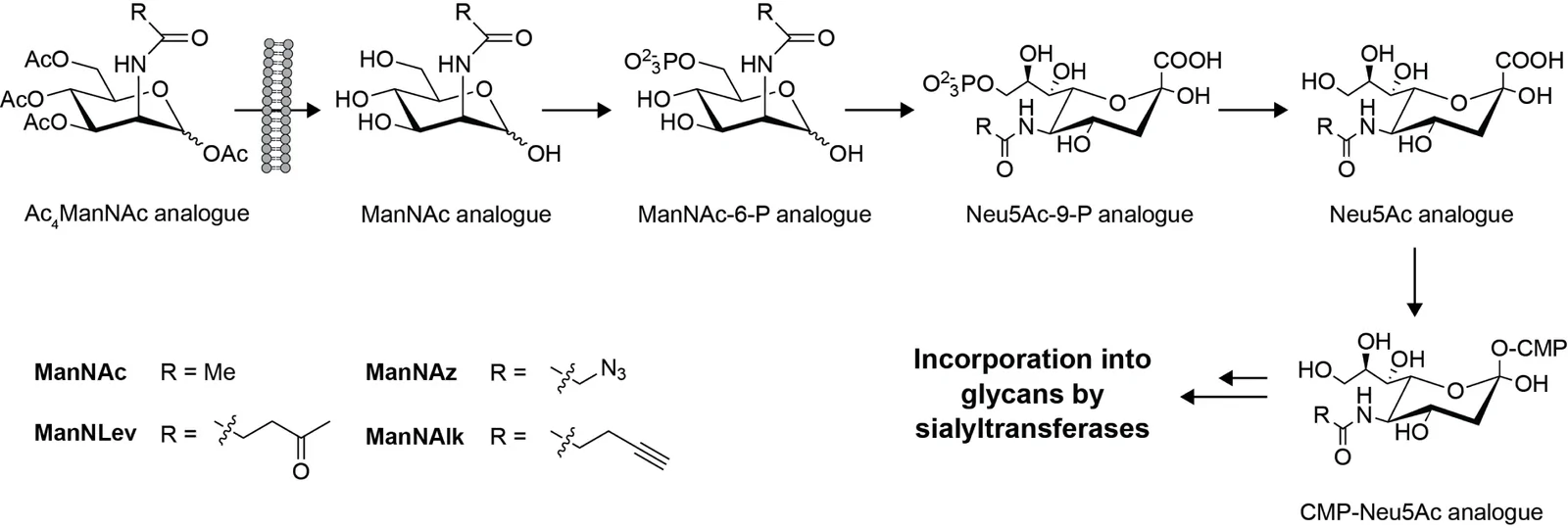
MOE on Sia ManNAc analogues are used by the sialic acid biosynthetic pathway and incorporated into glycans by sialyltransferases.

Sia are highly abundant monosaccharides that are often found at terminal positions on glycan chains. Since the biosynthetic machinery for their incorporation displays high promiscuity for chemical modifications, Sia were chosen for seminal MOE experiments in the field [25,26]. The terminal position of Sia on glycan chains means that they are easily detectable when tagged. Furthermore, being a terminal modification, there is no risk of the introduced tags interfering with subsequent glycosylation steps. The Reutter group discovered that *N*-acetylneuraminic acid (Neu5Ac) analogues with modified acylamide side chains were incorporated into glycans by the cellular biosynthetic machinery [23,24]. Radioactive versions of Neu5Ac precursors were incorporated into a rat cell line and *in vivo* in rats. Crucially, analogues of *N-*acetylmannosamine (ManNAc) were shown to be metabolised to Neu5Ac analogues via the intermediate ManNAc-6-phosphate derivative (Figure 1). Follow-on studies elucidated the different roles of Sia, such as the regulation of cell–cell-dependent growth and their importance in virus–receptor interactions [27,28].

The first use of chemoselective ligation on modified Sia precursors came in the following years from the Bertozzi group using ketones as a chemical handle [29,30]. Ketones are not present on naturally occurring biomolecules on the cell surface and can be derivatised with hydrazide-based probes under physiological conditions. The group used an *N-*levulinoyl mannosamine (ManLev) precursor that is accepted by the sialic acid biosynthetic pathway and metabolised to SiaLev (Figure 1). Once incorporated onto cell surface proteins on human cell lines, chemoselective ligation was performed with biotin-hydrazide to analyse chemically tagged cells. Sarkar et al. found that acetyl protecting groups increased the hydrophobicity of monosaccharides and were removed by non-specific esterases (NSE) in the cytosol, improving membrane permeability and increasing cellular uptake [31]. This strategy allowed for chemical cell surface tagging at lower precursor concentrations of Ac_4_ManLev (250-fold), compared with ManLev [32]. The biosynthesis of different keto-ManNAc analogues was tested by the Bertozzi group. Ac_4_ManNAc analogues with ketones and varying lengths of *N-*acyl side chains were tested for their metabolic efficiency [33]. Increasing *N-*acyl side chain length led to decreasing levels of free sialic acid, indicating that assessing the metabolic fate of chemically tagged monosaccharides is necessary.

Chemoselective ligation using ketones laid the foundation for the development of bioorthogonal reactions of modified Sia. Although ketones are not naturally present on the cell surface, intracellular ketones compete with keto-metabolites [30]. Furthermore, ketone-hydrazide condensation requires a pH of 5–6 to achieve optimal rates, which is not conducive to *in vivo* derivatisation under physiological conditions. The Staudinger–Bertozzi ligation occurs between a phosphine and an azide and allows for faster and more efficient reactions at least compared with hydrazide chemistry [34]. Phosphines and azides are not endogenous to cells, neither intra- nor extracellularly. The Bertozzi group tagged ManNAc with an azide (ManNAz) to be converted to the Neu5Ac analogue Neu5Az inside cells and traced by the Staudinger–Bertozzi ligation [35]. Azides began to be widely used to investigate the role of individual monosaccharides on glycoproteins and of other biomolecules [36–39]. Successful application of the Staudinger–Bertozzi ligation in various cell lines was a precursor to the first *in vivo* experiments [40]. Mice were injected with Ac_4_ManNAz followed by phosphine-FLAG. Splenocytes were then isolated, and labelled cells were detected by flow cytometry. This study was a proof-of-concept of the first bioorthogonal reaction in animals, pointing to different levels of endogenous glycoproteins in various mouse tissues. A traceless version of the Staudinger–Bertozzi ligation followed in which no trace of the oxidised phosphine remained in the biomolecule, allowing applications in peptide synthesis [41].

While the Staudinger–Bertozzi ligation was being applied *in vivo*, the groups of Sharpless and Meldal reported a Cu(I)-catalysed azide-alkyne cycloaddition reaction, or CuAAC [42,43]. Sharpless had defined the term ‘click reactions’ as a set of facile transformations to chemoselectively fuse molecules together [44]. The groups realised separately that a Cu(I) catalyst was a substantial improvement in the rate and regioselectivity of the classical non-catalysed 1,3-dipolar cycloaddition between azides and alkynes described by Huisgen [45]. CuAAC was then applied to glycans [46]. However, the Cu(I) catalyst proved to be cytotoxic, preventing the use of the reaction in living systems at the time. Furthermore, Cu(I) alone is unstable in solution. This issue would be addressed by the development of ligands for the Cu(I) species, some of which are membrane-impermeable and trap the catalyst outside the cell [47,48]. In 2008, the Bertozzi group reported a Cu-free click reaction now known as strain-promoted azide-alkyne cycloaddition (SPAAC) [49]. SPAAC occurs between an azide and a strained alkyne, such as a cyclooctyne, and does not require a catalyst [50]. The cyclooctyne ring strain leads to a lower activation barrier for alkyne deformation, accelerating the uncatalysed reaction [50,51]. Electron-withdrawing substituents further aid in lowering the energy of the lowest unoccupied molecular orbital. For instance, kinetic analyses by Baskin et al. of SPAAC using a cyclooctyne with a difluoromethylene moiety (DIFO) showed improved rate constants of between one and two orders of magnitude compared with the Staudinger–Bertozzi ligation and other strain-promoted cycloadditions [52]. SPAAC was then successfully used to study the dynamics of glycoprotein trafficking from the Golgi apparatus to the cell surface with live-cell imaging. Subsequently, SPAAC was employed *in vivo* in mice injected with Ac_4_ManNAz followed by DIFO-cyclooctyne-FLAG to study the glycoproteome of various mouse tissues [53]. In zebrafish models, SPAAC served to evaluate glycosylation changes during embryonal development [54].

Following the first SPAAC applications, cycloadditions with alternative 1,3-dipoles such as nitrones or nitrile imides were developed, some of which are light-activatable [55,56]. The primary goal of these efforts was to increase ligation kinetics—although much faster than Staudinger–Bertozzi ligations, SPAAC reactions were still typically slower than CuAAC reactions [57]. Perhaps the most prominent example of a modern, fast bioorthogonal ligation is the tetrazine ligation that employs inverse electron-demand Diels–Alder reactivity. Originally developed by the Fox group, tetrazine ligation occurs between an electron-poor tetrazine and an electron-rich dienophile such as norbornene, *trans*-cyclooctene, or cyclopropene [58]. This reaction has become prominent in the field of bioconjugation due to its bioorthogonality and very fast reaction kinetics in water, typically with second-order rate constants that are orders of magnitude higher than those of SPAAC reactions [58,59]. A host of new bioorthogonal reactions with different properties have been developed since. For more detailed accounts of these, we refer to existing reviews [57,60–66]. This work encompasses the establishment of pathways for cellular incorporation of chemically modified monosaccharides. We note that a large body of work has established the direct chemical modification of cell surface glycans by exo-enzymatic labelling that is covered elsewhere [67].

## Roadblocks in the pathway for expansion of MOE to different sugars

**Figure 2:**
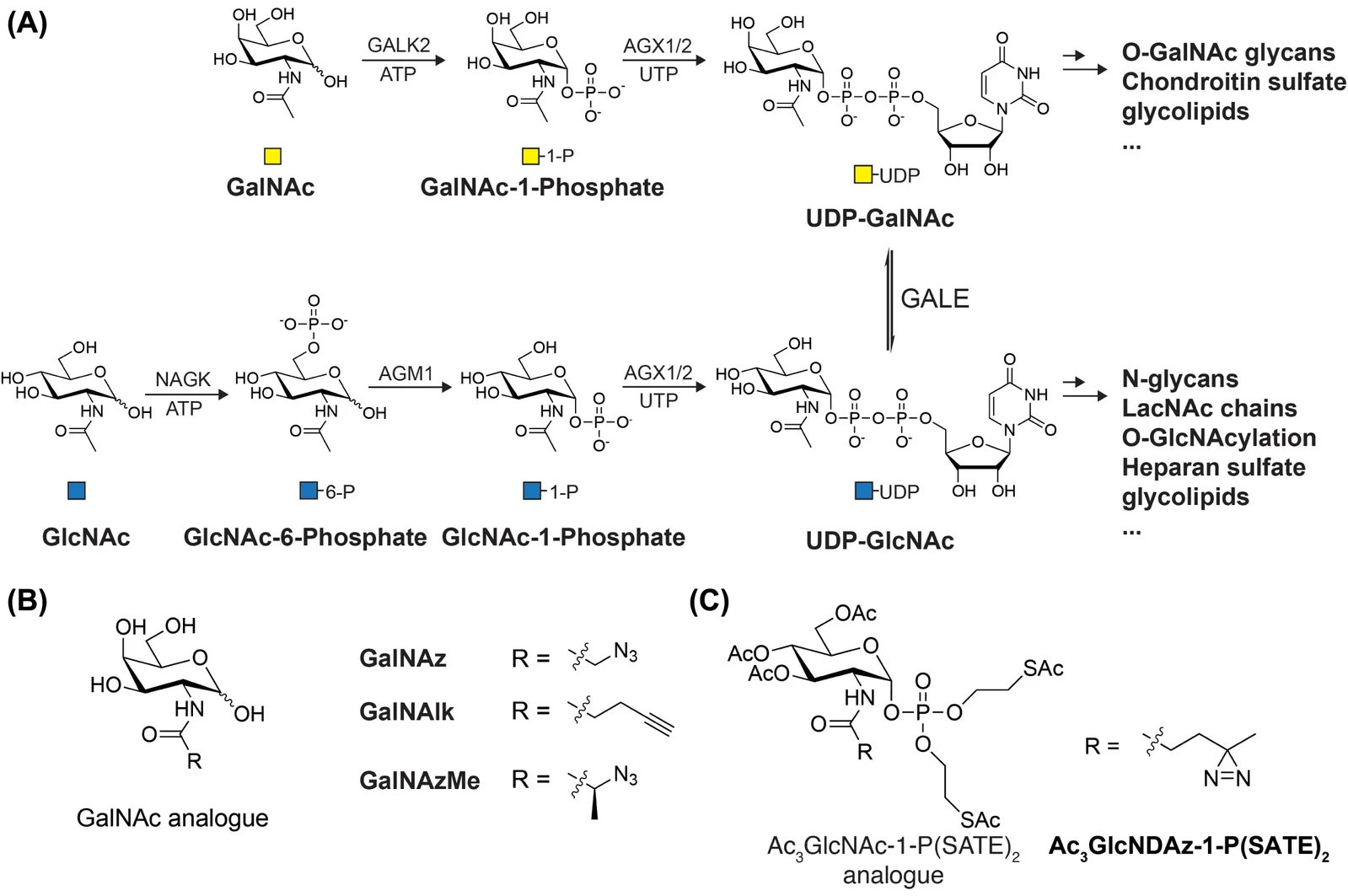
The GalNAc and GlcNAc salvage pathways and GalNAc/GlcNAc analogues (**A**) GalNAc is sequentially converted to GalNAc-1-P by GALK2 and then to UDP-GalNAc by AGX1/2. GlcNAc is converted to GlcNAc-6-phosphate by NAGK, then to GlcNAc-1-phosphate by AGM1, and finally to UDP-GlcNAc by AGX1/2. (**B**) UDP-GalNAc and UDP-GlcNAc are interconverted by GALE. UDP-GalNAc and UDP-GlcNAc are essential for the biosynthesis of a wide variety of abundant glycans. (**C**) Chemical structure of caged modified sugar-1-phosphate Ac_3_GlcNDAz-1-P(SATE)_2_.

While Sia are the most amenable monosaccharides to test new bioorthogonal reactions in cells, the principle was rapidly expanded to GalNAc and GlcNAc (Figure 2B) [68,69]. The GlcNAc salvage pathway uses the kinase NAGK that converts GlcNAc to GlcNAc-6-phosphate, with subsequent isomerisation to GlcNAc-1-phosphate by phosphohexomutase AGM1 and condensation with UTP to UDP-GlcNAc by the phosphorylases AGX1/2 (Figure 2A) [70–73]. UDP-GlcNAc can be used by a host of cellular GTs or interconverted to UDP-GalNAc by the UDP-galactose-4′-epimerase GALE [74]. UDP-GalNAc can also be biosynthesised through the enzymes of the GalNAc salvage pathway [75,76]. Here, the kinase GALK2 generates GalNAc-1-phosphate that is then converted to UDP-GalNAc by AGX1/2 (Figure 2A).

In seminal work, the acetamide in GlcNAc and GalNAc was replaced with an azidoacetyl moiety to furnish GlcNAz and GalNAz, respectively. Vocadlo et al. found that GlcNAz was not as abundantly incorporated into cell surface glycans as expected, prompting investigation into the possibility that GlcNAz might not be as efficiently converted to the modified nucleotide sugar (UDP-GlcNAz) by the GlcNAc salvage pathway [68]. *In vitro* kinetic studies on AGM1 and AGX1 revealed that reaction rates remain comparable between GlcNAc and GlcNAz substrates. However, AGX1 was identified as a bottleneck for biosynthesis of UDP-GlcNAz. It was also confirmed that the modified UDP-GlcNAc is accepted by OGT and used for glycosylation of cytoplasmic proteins. It was concluded at the time that UDP-GlcNAz is made and used particularly by OGT in the cell [68].

Incorporation of an azide moiety into GalNAc led to the monosaccharide GalNAz that was accepted by the native GalNAc salvage pathway [69,77]. Competition experiments in Chinese hamster ovary (CHO) cells revealed that after feeding with Ac_4_GalNAz, cell-surface incorporation could be completely suppressed in the presence of excess GalNAc, but not by Gal, demonstrating the uptake of GalNAz by the GalNAc salvage pathway [69]. In addition, the interconversion between UDP-GalNAz and UDP-GlcNAz by GALE was studied by feeding CHO cells either Ac_4_GalNAz or Ac_4_GlcNAz. At the time, no azido signal from cell surface proteins was identified from cells fed with Ac_4_GlcNAz, suggesting that UDP-GalNAz and UDP-GlcNAz were not interconverted. However, further investigation by Boyce et al. later showed that feeding cells with Ac_4_GalNAz resulted in better incorporation into O-GlcNAc glycosylated proteins than even Ac_4_GlcNAz [78]. By extracting and analysing cellular UDP-sugars as a relatively new application, it was found that effective interconversion happened between UDP-GlcNAz and UDP-GalNAz. AGX2 was identified as the rate-limiting biosynthetic step in the formation of UDP-GlcNAz from Ac_4_GlcNAz. Opting to swap reactive groups for CuAAC, alkyne-tagged monosaccharides were also developed [53]. The Pratt group used Ac_4_GlcNAlk and Ac4GalNAlk to chemically tag glycoproteins in human cell lines and attributed differences in incorporation of the modified sugar to varying efficiencies in the biosynthesis of UDP-GlcNAlk [77,79].

The interconversion between analogues of UDP-GalNAc and UDP-GlcNAc by GALE limits the investigation of specific types of glycosylation. Therefore, developing approaches to prevent the activity of GALE is of great interest. Deletion or knockdown of *GALE* results in major consequences: an imbalance in sugar nucleotides causes dramatic global changes in glycosylation patterns, affecting associated functions such as cell signalling to an extent that is usually lethal in whole organisms [80–84]. While this can provide invaluable information for studying diseases such as galactosemia, it does not provide the best model system to study endogenous cellular glycosylation. However, a handful of human cell lines with GALE KO have been created, including CHO (CHO *ldlD*), K562, and HEK293T, allowing the separation of cellular UDP-sugar pools. Another way of thwarting the activity of GALE is by the design of the modified sugars themselves. Analysing an existing co-crystal structure of GALE and UDP-GalNAc, we found that the acetamides of UDP-GlcNAc/GalNAc are bound in long and narrow binding pockets that leave little room for bulky modifications [85,86]. A range of UDP-GalNAc analogues were tested *in vitro* using recombinant human GALE, and epimerisation to UDP-GlcNAc analogues was evaluated by HPLC. All compounds containing a branched side chain were not efficiently epimerised by GALE. We identified a branched analogue, UDP-GalNAzMe (Figure 2B), that was not interconverted into the corresponding UDP-GlcNAc analogues but still incorporated into the cellular glycoproteome by GalNAc transferases. This finding marked the development of the first O-GalNAc glycosylation-specific molecular probe, even without genetic disruption of GALE expression [87].

## An artificial biosynthetic pathway for sugar analogue activation

**Figure 3:**
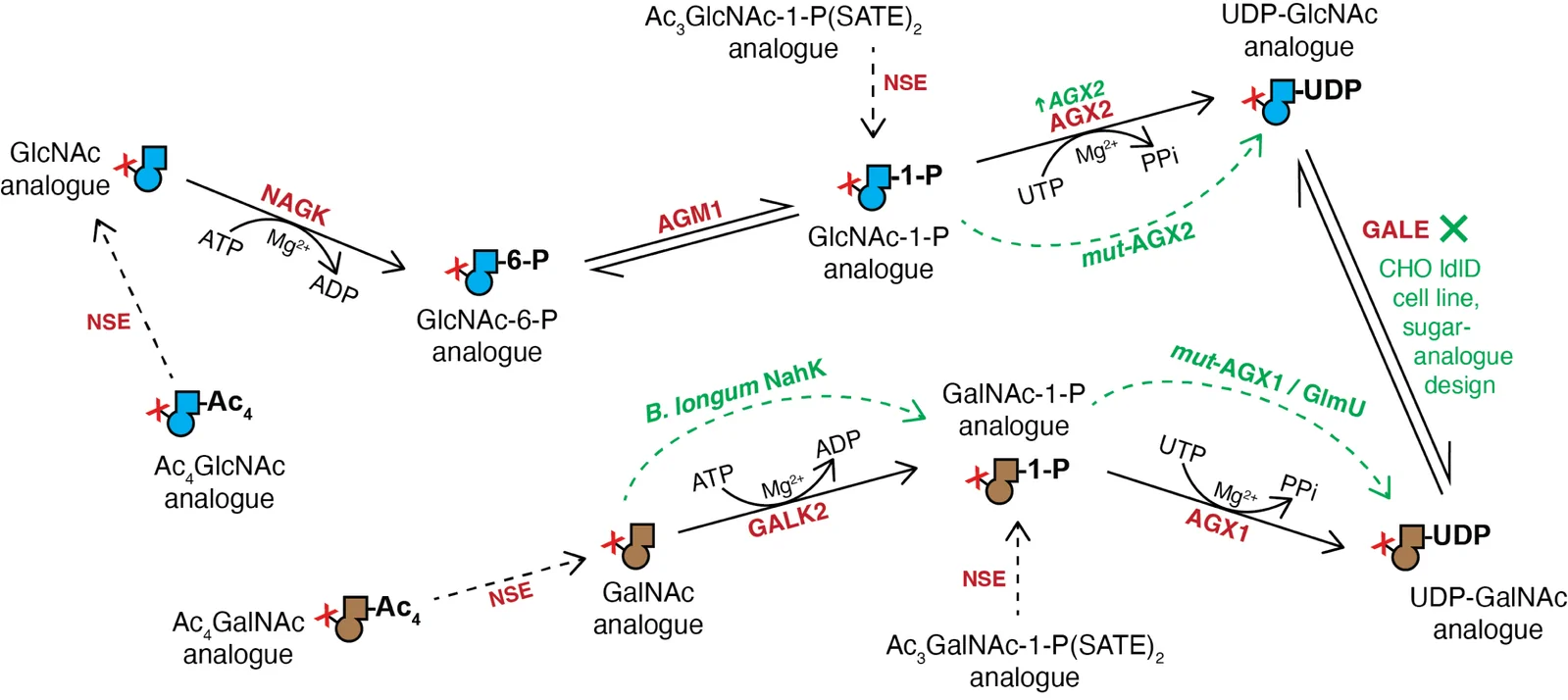
UDP-GlcNAc and UDP-GalNAc biosynthetic pathways Endogenous enzymes are indicated in red, while methods of bypassing the native biosynthesis steps are indicated in green. NSE are present in the cytoplasm and remove acetyl groups.

With the development of an increasing number of bioorthogonal monosaccharides came the realisation that salvage pathways show varying efficiencies in using them. While a wide range of modifications was tolerated on ManNAc analogues, modifications on GlcNAc and GalNAc were found to be less tolerated [88]. Inefficient biosynthesis makes these analogues more challenging to reliably incorporate into glycoproteins, sparking attempts to bypass these pathways. To test the conversion of monosaccharide derivatives to GlcNAc-1-phosphate and GalNAc-1-phosphate derivatives, the Wang group used the promiscuous kinase NahK *in vitro* (Figure 3) [89,90]. NahK showed particular tolerance to *N-*acyl (C2) modifications of GlcNAc, while modifications at C3 and C6 were not well tolerated by the enzyme. The inactivity of NahK with galactose (Gal) derivatives indicated that the 2-amido group is required for enzymatic activity. Further work by the same group tested the activity of the bacterial pyrophosphorylase *N*-acetylglucosamine-1-phosphate uridyltransferase (GlmU) with a panel of GlcNAc-1-phosphate and GalNAc-1-phosphate analogues [91]. Modifications to the C6 positions of both epimers were moderately well tolerated by GlmU. Additionally, the enzyme showed good acceptance of small *N*-acyl (C4) substituents on GlcNAc-1-phosphate, but conversion decreased dramatically for larger substituents. By contrast, GlmU did not accept any *N*-acyl modifications of GalNAc-1-phosphate, possibly due to steric clashes of this group at the GlmU active site. Finally, comparison of human AGX1 and *Escherichia coli* GlmU activities with analogues of GlcNAc-1-phosphate and GalNAc-1-phosphate demonstrated that AGX1 shows a substantially better acceptance of *N*-acyl modifications and slightly better C4 and C6 modification tolerance than GlmU (Figure 3) [91].

To test the efficiency of incorporation of sugars with varying *N*-acyl modifications, the Piller group treated modified CHO ldlD cells that lack GALE activity with a panel of GalNAc analogues [75,76]. Cell-surface analogue incorporation was quantified using a fluorescein-labelled GalNAc-specific lectin and detected using flow cytometry. *N*-acyl groups containing azido or hydroxy groups demonstrated the greatest uptake into cell-surface glycans, while other groups, such as *N-*propionyl and *N-*formyl substitutions, showed substantially lower incorporation.

Studies into developing photo-crosslinkable monosaccharide analogues by the Kohler group aimed to bypass the first two steps of UDP-GlcNAc biosynthesis [92–94]. Cells were fed Ac_3_GlcNDAz-1-P(Ac-SATE)_2_, a caged, plasma-membrane-permeable compound that is subsequently deprotected in the cytosol by NSE (Figure 2C). Instead of the expected biosynthesis of UDP-GlcNDAz by AGX1/2, it was found that AGX1/2 enzymes were not able to act on this bulkier *N-*acyl-modified sugar. It was hypothesised that the hydrophobic pocket that naturally accommodates the GlcNAc/GalNAc acetamide in the active site of AGX1, interacting with residues F381 and F383, is likely too small to accept the diazirine. Therefore, a human AGX1^F383G^ variant enzyme was generated with a larger active site pocket that is able to biosynthesise UDP-GlcNDAz [78]. The ensuing use of engineered, promiscuous AGX1 variants by labs around the world highlighted the success of this approach. Testing a panel of AGX1 single and double mutants, the F383G and F383A mutants were found to efficiently convert GalNAc-1-phosphate analogues with linear alkynyl side chains into their UDP-sugar counterparts [95]. The Chen group engineered AGX2 instead of AGX1, boosting biosynthesis of UDP-GlcNAlk and allowing MOE in mice [96]. We employed the AGX1^F383A^ variant to biosynthesize UDP-GalNAzMe from a caged GalNAzMe-1-phosphate. In contrast, the F383G variant was not suitable for the same application, highlighting a need for careful adjustment of biosynthesis depending on the monosaccharide analogue in use. We have since expanded the use of these enzymes to the chemoenzymatic synthesis of various analogues of UDP-GalNAc and UDP-GlcNAc [97–99].

Bypassing earlier stages of UDP-sugar biosynthesis using caged phosphate analogues is a viable and useful technique. However, the synthesis of these compounds is laborious. Our recent work has addressed these drawbacks by bringing together a cohesive in-cell artificial biosynthetic pathway for GalNAc analogues. Stably transfecting cells with plasmids encoding both NahK and engineered human AGX1^F383A^ produced a highly efficient pathway for the biosynthesis of UDP-GalNAlk and 6-carbon hexynoate-armed UDP-GalN6yne, as well as several azide-containing analogues [87] (Figure 2B). The NahK-AGX1^F383A^ biosynthetic pathway was thus instrumental in the application of novel chemical tools, also offering the availability of control cell lines that lack the enzymes of the biosynthetic pathway. Such control samples are invaluable to confirm that the observed fluorescence signal is indeed caused by enzymatic glycosylation, a crucial reference sample in the application of new tagged monosaccharides.

## Towards precision tools to probe the activity of O-GalNAc glycosyltransferases

**Figure 4:**
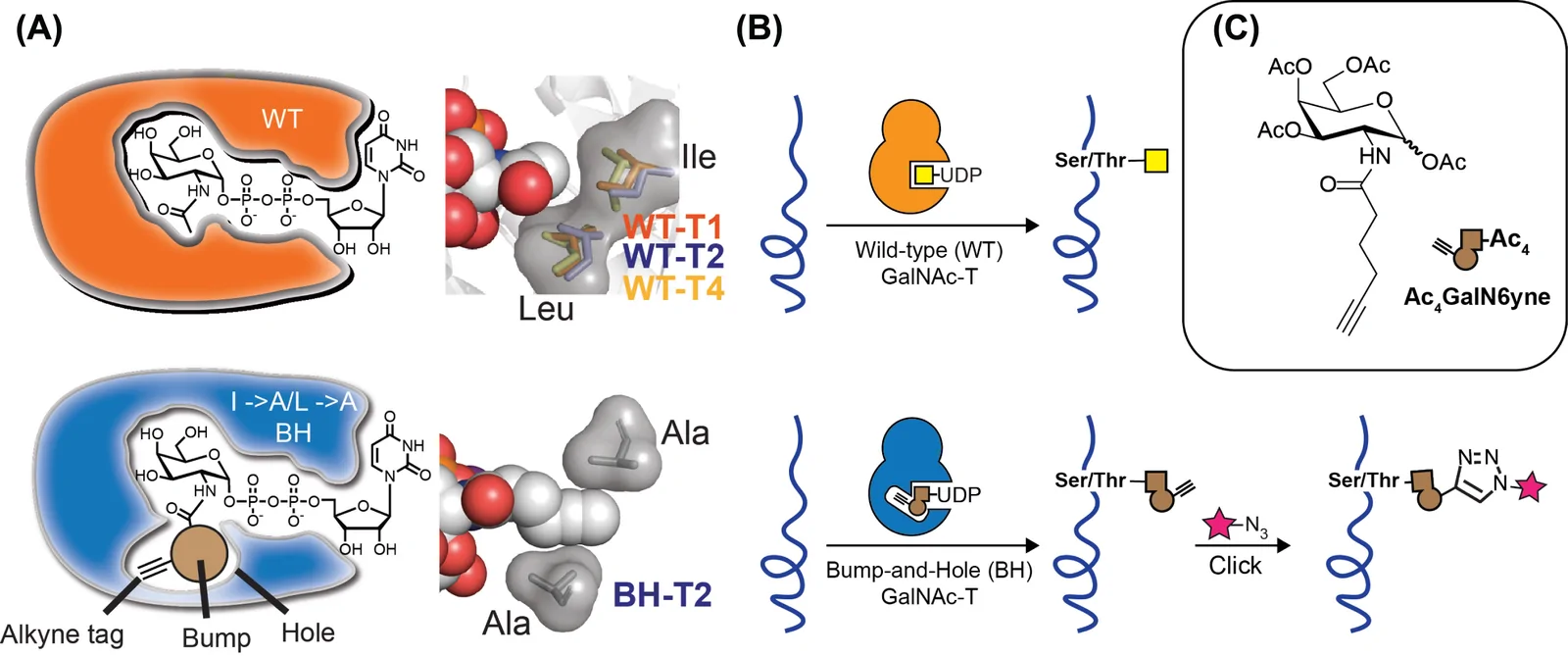
BH engineering on GalNAc-Ts (**A**) Wild-type (WT) GalNAc-Ts bound to UDP-GalNAc and BH-GalNAc-T2 bound to a UDP-GalNAc analogue. Crystal structures show WT-GalNAc-T1 (PDB: 1XHB), WT-GalNAc-T2 (PDB: 4DOT) and WT-GalNAc-T4 (PDB: 6H0B) bound to UDP-GalNAc, and BH-GalNAc-T2 bound to UDP-GalN6yne (PDB: 6NQT, bottom). Gatekeeper residues Ile and Leu are mutated to Ala to generate a ‘hole’ in the enzyme. (**B**) The GalNAc analogue can be traced on substrates using CuAAC to add a chemical tag (pink star) for downstream analysis. (**C**) Chemical structure of peracetylated modified sugar Ac_4_GalN6yne. Figure re-printed in part from [95].

Ever since the first development to decipher protein-ligand interactions by Schreiber and the application to kinase inhibitors by Shokat, the bump-and-hole (BH) tactic has been a cornerstone of innovation in chemical biology [100,101]. The BH tactic features engineering the active site of a protein to contain a ‘hole’ complementary to a ‘bump’ on a modified ligand while retaining much of the catalytic activity and/or biological function of the protein [100,102]. Since these early innovations, the BH approach has been applied to investigate a range of transferase enzymes, including methyltransferases, ADP-ribosyltransferases, palmitoyltransferases, and glycosidases [103–107]. Qasba, Hsieh-Wilson and colleagues were the first to engineer a GT to accept a bioorthogonal UDP-sugar analogue, using the galactosyltransferase B4GALT1 as an *in vitro* glycosylation tool [108]. The Bertozzi group then developed the BH approach systematically for the enigmatic GalNAc transferase family, eventually allowing the identification of protein substrates of individual GalNAc-Ts [95,109]. Crystal structures of GalNAc-T2 identified hydrophobic so-called gatekeeper residues, Ile, Leu, and Phe, bound to UDP-GalNAc [110]. These are highly conserved residues across the GalNAc-T family [111,112]. Replacing the Ile253 and Leu310 residues in GalNAc-T2 with smaller Ala residues led to accommodation of the elongated *N-*acyl side chain in modified UDP-GalNAc analogues (Figure 4A) [109]. The double mutant I253A/L310A was identified to be selective (>100:1 incorporation) for bumped UDP-GalN6yne over native UDP-GalNAc. The double mutant retained the peptide specificity and much of the kinetic properties compared with the WT enzyme, with some changes in *K_M_* and *k_cat_* depending on the substrate. The use of UDP-GalN6yne with the engineered enzyme allows for derivatisation with a clickable chemical tag, such as fluorophores or biotin probes (Figure 4B,C).

Since GalN6yne is not normally accepted by the enzymes of the GalNAc salvage pathway (see above), implementation of the BH-GalNAc-T approach in human cells required engineered biosynthesis. An early version of a cellular GalNAc-T BH tactic involved the use of a caged GalN6yne-1-phosphate. The gene encoding the AGX1^F383A^ variant was included on the same plasmid as the engineered *GALNT* gene to drive both biosynthesis and glycosylation in the same cell [95]. Differential MS (glyco)proteome analysis was employed to yield, for the first time, both substrate proteins and glycosylation sites of different GalNAc-T isoenzymes in a gain-of-functionality approach. The BH tactic allowed site resolution of glycosylation in the substrate apolipoprotein AI. GalNAc-T1 was found to unambiguously glycosylate Thr221 on ApoAI in cells, while GalNAc-T2 glycosylated Ser225, as a crucial complement to previously described MS data using *GALNT* KO cells [15].

The use of BH-GalNAc-Ts provided a new view into O-GalNAc glycosylation but was still reliant on a caged GalN6yne-1-phosphate reagent. In an effort towards a more readily available precursor to UDP-GalN6yne, the gene encoding *B. longum* NahK was added to the plasmids in a second generation of the tactic (see above). Implementation of this enhanced BH system in living cells allowed much more straightforward cellular application [87]. As an extension of this work, our group demonstrated, shortly after Chen and colleagues, cell specificity of biorthogonal glycan tagging in co-culture systems and even in mice [62,113]. Established in only one cell line among a co-culture system, engineered biosynthesis allowed UDP-sugar biosynthesis only in that cell line. Both cellular and secreted glycoproteins were chemically tagged and could be distinguished from the respective other cell lines. This work allowed us to distinguish between the glycoproteomes of murine tumour cells, expressing NahK/AGX1^F383A^/BH-GalNAc-T2, and human mock-transfected host cells in an *in vitro* co-culture model of a tumour microenvironment. MS-based proteome analyses measured only murine protein substrates of GalNAc-T2 commensurate with the setup of the assay [113]. We termed this method bioorthogonal cell-specific tagging of glycoproteins (BOCTAG). The biosynthetic pathway was subsequently used by Kohler, Fox, and colleagues to deliver a tetrazine-tagged GlcNAc analogue [114].

## Extension of BH engineering to other GT families

Proteoglycans carry long glycosaminoglycan (GAG) chains that are essential for tissue development and homeostasis, cell signalling, and structural scaffolding of the extracellular matrix. The most common GAGs are heparan sulfate (HS) and chondroitin sulfate (CS) [115]. Both HS and CS chains begin with a common tetrasaccharide linker sequence: xylose (Xyl), two Gal residues, and glucuronic acid (GlcA) in the GlcAβ(1-3)Galβ(1-3)Galβ(1-4)Xylβ-Ser sequence. The initial addition of O-linked Xyl is catalysed by xylosyltransferases XT1 and XT2 [116,117]. To investigate the proteoglycan substrates of XT1 and XT2, we established the BH system on both these enzymes [118]. A previous study by the Huang group found that WT-XT1 had a 20-fold lower activity towards a modified nucleotide sugar, UDP-6AzGlc, compared with native UDP-Xyl [119]. We identified conserved gatekeeper residues in XT1 (Trp392, Leu525, and Leu526) and in XT2 (Trp298, Leu431 and Leu432) that are in proximity to C5 of UDP-Xyl. We found that a single amino acid variant, L526G, of XT1 (BH-XT1 from here on) displayed a 7–8-fold preference for UDP-6AzGlc compared with UDP-Xyl or UDP-Glc. To establish the new BH system in cells, biosynthesis of the modified nucleotide sugar had to be ensured. Although human cells do not have a salvage pathway for UDP-Xyl, we were able to leverage the native biosynthetic pathway for UDP-Glc by using the modified sugar 6AzGlc. In the biosynthetic pathway of UDP-Glc, Glc is first phosphorylated to Glc-6-phosphate by hexokinase, then isomerised to Glc-1-phosphate by phosphoglucomutase, and finally converted to UDP-Glc by the pyrophosphorylases UGP1/2. However, due to the existence of an azide moiety on C6, phosphorylation by hexokinase is not possible. To circumvent this incompatibility, we used a caged 6AzGlc-1-phosphate precursor for cell feeding. Instead of a SATE-caged modified sugar-1-phosphate, we fed cells with a membrane-permeable phosphoramidate diester-modified caged sugar-1-phosphate. Using the BH system, we identified proteoglycan substrates specific to XT1 and XT2.[118].

Dysregulation of *N*-glycosylation is associated with CDG and linked to various neurological and developmental disabilities. *N-*glycans are pre-assembled as a lipid-linked oligosaccharide in the endoplasmic reticulum and then transferred en bloc to proteins [120]. The glycan branch is then processed in the Golgi by glycosidases and GTs. A number of GlcNAc transferases called MGATs modify *N-*glycan cores towards extended structures. Among these, MGAT1 is a crucial bifurcation point between oligomannose and hybrid/complex structures [121]. MGAT5 is responsible for the addition of GlcNAc to the Manα(1-6)Man arm to generate the GlcNAcα(1-6)Man structure that is frequently associated with cancer [122]. Hannover and colleagues recently established a tosylated, alkyne-containing GlcNAc derivative as a selective tool for hybrid *N-*glycans [123]. The authors found that the GlcNAc analogue MM-JH-1 entered *N-*glycans rather than, for instance, cytosolic O-GlcNAc glycans. Through extensive validation in imaging and MS, it was found that MGAT1 appeared to transfer MM-JH-1 to *N-*glycan cores and, surprisingly, retained a propionate caging group at the 3-position.

We established BH systems for MGAT5 and for MGAT1 [97,99]. Taking inspiration from our previous work on GalNAc transferases, gatekeeper residues were identified close to the GlcNAc acetamide and mutated to smaller amino acids to generate holes in MGAT5 and MGAT1. Single and double variants were tested *in vitro* against a panel of modified UDP-GlcNAc analogues [98]. The BH pairs were established *in vitro*, initially employing the nucleotide-sugar UDP-GlcNButAz with BH-MGAT5 (double mutant F458V/F517L) and BH-MGAT1 (single mutant L267A). The structure–activity relationship on BH-MGATs was of particular interest: Instead of gaining new activity for bumped UDP-GlcNButAz, it was found for both enzymes that orthogonality was driven by the engineered BH-enzymes losing activity for UDP-GlcNAc. Competition experiments revealed that the WT-enzymes, while theoretically accepting UDP-GlcNButAz, preferred UDP-GlcNAc when both were provided at concentrations typically observed in cells. However, we noted that delivering UDP-GlcNButAz to living cells led to considerable background bioorthogonal signal, presumably due to the acceptance by more promiscuous GlcNAc transferases such as MGAT2 [94]. Neural network-driven molecular dynamic simulations were then employed to design a further refined, bumped substrate for MGAT1. Eventually, an isomeric compound termed UDP-GlcNPrAzMe was designed that exhibited less non-specific background incorporation. Cellular delivery of UDP-GlcNPrAzMe led to the direct observation of MGAT1 substrate with a reasonable signal-to-background ratio [99]. These findings confirmed the relevance of carefully designing bioorthogonal tags.

## Design rules for MOE

**Figure 5:**
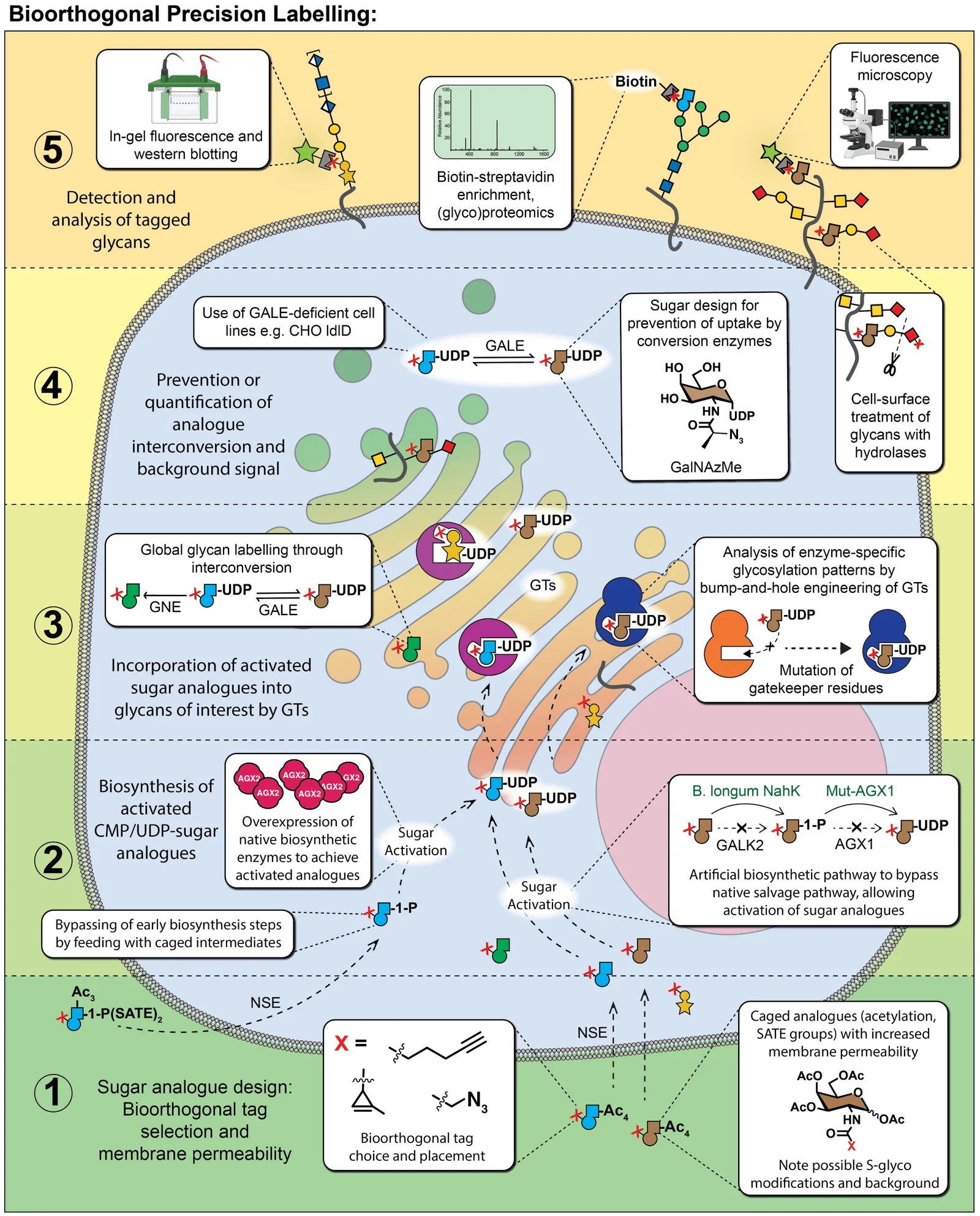
Experimental design rules for MOE In order for efficient labelling of glycans, the appropriate sugar analogue must be selected with consideration for the intended target glycans and the method of analysis. Biosynthesis of the appropriate activated sugar analogue by the relevant enzymes can be achieved through various methods and must then be verified. Following this, GTs of interest should be able to accept the activated sugar analogues, either in their WT state or through BH engineering. Background labelling and interconversion must be considered in the experimental design by either preventing certain forms of interconversion altogether or by removing interconversion products before analysis. Finally, the appropriate analysis method can be employed to investigate glycosylation. GNE: UDP-GlcNAc-2-epimerase/ManNAc Kinase.

With MOE approaches developed over the years, it has become apparent that certain design rules should be followed to assure cellular glycan incorporation and prevent unwanted side reactions (Figure 5).

1. **Choice of an appropriate monosaccharide:** We tend to consider first incorporation into the glycoproteome: choose which monosaccharide is targeted and what the chemical modification should be. If specific chemistry is needed, such as photo-crosslinking or SPAAC, the appropriate functional group should be chosen. Along with these considerations, it should be considered that the monosaccharide should be used by the GT(s) of choice after nucleotide-sugar formation. Care should be taken which WT-GTs will use the modified substrate or whether BH-engineering is required. The latter approach has the benefit that glycosylation can be rendered enzyme-specific but requires protein engineering and *in vitro* evaluation.
2. **Confirmation of a biosynthetic pathway:** As a second step after choosing the right monosaccharide, it is crucial to confirm that a biosynthetic pathway exists to generate the corresponding nucleotide-sugar analogue. While certain sugars will be accepted by native salvage pathways (e.g. most ManNAc analogues, GalNAz, and GlcNAz), others will require an artificial biosynthetic pathway that is appropriately designed to accept the sugar modifications (e.g. NahK and mutant AGX1 for bulkier GalNAc and GlcNAc analogues, including GalNAlk and GlcNAlk) [124]. The biosynthesis of activated nucleotide-sugar analogues must be directly confirmed, for instance, through characterizing cellular extracts by analytical methods such as high-performance anion exchange chromatography or MS.
3. **Caging groups:** As a third consideration, caging groups should be chosen to render the monosaccharide membrane-permeable. Per-acetylated analogues are efficiently uncaged but lead to non-specific background incorporation at high concentrations [125,126]. Propionyl esters as caging groups have been reported to circumvent these side reactions [123,127]. Phosphate caging groups may be more laborious to synthesize but can be employed as a useful means to deliver biosynthetic intermediates [86,92,95].
4. **Derivatisation:** Having designed delivery, biosynthesis, and glycosylation, the cellular incorporation of the modified sugar can be confirmed through derivatisation by bioorthogonal chemistry and addition of a reporter group of choice (e.g. biotin and fluorophore). Glycosylation can be profiled by in-gel fluorescence, flow cytometry, and MS, among other techniques.
5. **Quality control:** Treatment with specific enzymes can further prove incorporation into the desired glycan type—for instance, neuraminidases remove Sia, and mucinases remove mucin glycoproteins from cell surfaces, thereby reducing fluorescence signal from cell surfaces [113,124,128–130]. It may be important to remove any signal from potential incorporation of the modified sugar into other pathways and glycosylation types. For example, treatment with the peptide *N-*glycanase F can eliminate certain N*-*glycans from proteins in lysates, and *GALE*-KO cells can be used to prevent interconversion between analogues of UDP-GalNAc and UDP-GlcNAc. These methods can also be used to confirm whether the type of glycosylation observed is expected.
6. **Consideration of glycan elaboration:** Finally, we note that downstream elaboration of chemically modified monosaccharides is not fully evaluated yet. We and others have found that chemical tags are sometimes accepted by ensuing GT enzymes, although these findingsare not quantitative and not generalizable [87,95,118,131,132]. Similarly, acceptance of modified monosaccharides by glycosylation-processing enzymes can be impacted, as reported by Withers and colleagues [133]. We thus recommend carefully considering whether the targeted biological question is reliant on glycan elaboration—if so, the experimental design may require additional controls.

We recommend that these design rules be implemented in experimental design to obtain efficient, specific MOE tools with the desired effect.

MOE is an effective and versatile tool that can be harnessed to study glycobiology in a range of systems. While early developments saw the introduction of monosaccharide analogues into biosynthetic pathways for detection, newer applications that have come with the advancement of bioorthogonal chemistry have allowed for greater sensitivity and specificity of labelling techniques. Approaches such as BH engineering have allowed for increased precision of tools to study specific types of glycans. However, with rapid improvements in the selectivity and specificity of our current tools, there is scope for expansion of the repertoire of approaches.

In recent years, there has been an acceleration of pre-clinical and clinical initiatives in the field of glycobiology, such as the development of glycan-targeting chimaeras as a cancer therapeutic and the use of cell-surface glycan biomarkers for diagnostic purposes. Decades of research into glycosylation pathways have provided a strong foundation for these innovations, but challenges still prevail in the field. Emerging strategies in metabolic glycan labelling are focusing on minimising cellular perturbation and implementing labelling tools *in vivo* to provide insights into glycosylation pathways that remain understudied in more biologically relevant models.

Interdisciplinarity is important to the future of the field. For example, alongside the advancement of cutting-edge techniques in fields such as MS and super-resolution microscopy, there is may be opportunities for better understanding of aspects like glycan spatio-temporal dynamics.

Ultimately, these tools hold great potential to better our understanding of glycobiology, uncovering ample opportunities for exciting real-world applications.

## Abbreviations

BH: bump-and-hole
CS: chondroitin sulfate
CHO: Chinese hamster ovary
CDG: congenital disorders of glycosylation
DIFO: difluorocyclooctyne
Gal: galactose
GAG: glycosaminoglycan
GlcA: glucuronic acid
GTs: glycosyltransferases
HS: heparan sulfate
KO: knockout
GalNAc: *N*-acetylgalactosamine
GlcNAc: *N*-acetylglucosamine
ManNAc: *N*-acetylmannosamine
Neu5Ac: *N*-acetylneuraminic acid
ManLev: *N*-levulinoylmannosamine
ManNAz: *N*-azidoacetylmannosamine
MS: mass spectrometry
MOE: metabolic oligosaccharide engineering
NSE: non-specific esterases
Sia: sialic acids
SPAAC: strain-promoted azide–alkyne cycloaddition
OGT: UDP-GlcNAc:polypeptidyltransferase
Xyl: xylose
